# Effect of Anti-Müllerian Hormone on Oocytes In Vitro Maturation in Sheep

**DOI:** 10.3390/ijms27135701

**Published:** 2026-06-24

**Authors:** Peipei Zhang, Yupeng Li, Xiaodi Shi, Xiaofei Guo, Dawei Yao, Hui Sheng, Jinlong Zhang, Yuan Cai, Xiaosheng Zhang

**Affiliations:** 1 Tianjin Key Laboratory of Animal Molecular Breeding and Biotechnology, Tianjin Engineering Research Center of Animal Healthy Farming, Institute of Animal Science and Veterinary, Tianjin Academy of Agricultural Sciences, Tianjin 300381, China; 2 School of Life Science and Engineering, Northwest Minzu University, Lanzhou 730030, China

**Keywords:** anti-Müllerian hormone (AMH), sheep, oocytes, maturation, single-cell RNA sequencing

## Abstract

Improvement in the in vitro maturation (IVM) of oocyte quality is a gateway to enhancing the efficiency of in vitro embryo production. The anti-Müllerian hormone (AMH) is a crucial hormone secreted by granulosa cells that effectively suppresses primordial follicle recruitment and regulates follicular growth and development. This study was designed to investigate the role of AMH on the IVM of sheep oocytes. In this current study, oocytes in vitro were cultured in media supplemented with AMH. We comprehensively analyzed the impact of AMH on various developmental parameters of sheep oocytes, such as cellular activity, cortical granules (CGs) migration, cytoskeleton and mitochondrial function of oocytes. Furthermore, Smart-seq2 single-cell RNA sequencing (scRNA-seq) was employed to elucidate the oocytes’ development. The results showed that treatment with 100 ng/mL improved the maturation rate of the oocytes, the normal distribution rate of cortical granules and mitochondrial function, while reducing the rate of spindle abnormalities in oocytes. A total of 741 differentially expressed genes (DEGs) were observed between the FSH_12 h and AMH_12 h groups, and 746 DEGs were observed between the FSH_24 h and A+F groups. KEGG pathway analysis revealed that the FSH_12 h and AMH_12 h groups significant enrichment in DEGs were associated with p53, MAPK, PI3K-Akt and TGF-beta signaling pathways, and the FSH_12 h and AMH_24 h groups significant enrichment in DEGs were associated with cAMP, AMPK, Hedgehog and estrogen signaling pathways. These findings suggest that AMH may regulates oocytes IVM via several candidate signaling pathways. Our results provide preliminary clues for exploring the regulatory mechanism of sheep oocyte maturation and optimizing relevant culture systems.

## 1. Introduction

In mammals, the developmental state and maturation quality of oocytes determine whether early embryos can develop normally [1,2]. In vitro maturation (IVM) of oocytes is the key to in vitro embryo production, which can provide abundant oocyte resources for in vitro fertilization and somatic cell nuclear transfer [3,4], and the study of its maturation mechanism is important for improving the maturation efficiency and in vitro embryo production [5]. In multiple species, the quality and efficiency of oocytes matured in vitro were reduced compared to those matured in vivo, with blastocyst rates close to 80% for in vivo mature oocytes and mostly 35% for in vitro mature oocytes [6].

The anti-Müllerian hormone (AMH) is a 140 kDa dimeric glycoprotein of the transforming growth factor beta (TGF-β) family, produced by ovarian follicular granulosa cells [7,8]. In females, AMH is an important regulator of ovarian production and is predominantly expressed in the gonads [9,10]. AMH is one of the indicators of ovarian reserve function and also serves as a predictor of oocyte yield following controlled ovarian stimulation [11]. The serum AMH levels reflect the number of activatable luminal follicles in the ovary, as well as the size of luminal follicles [12,13,14]. AMH plays an important role in the recruitment of primordial follicles and in the selection of sinusoidal follicles as they develop into mature follicles [15,16]. AMH inhibits primordial follicle recruitment into the growing follicle pool in a negative feedback pathway [17,18], while also inhibiting the selection of dominant follicles by modulating the follicle-stimulating hormone (FSH) sensitivity of the luminal follicles [13,19].

Regarding the effect of AMH on oocyte maturation, Takahashi found that AMH inhibited oocyte meiosis in rats, suggesting that AMH may be an inhibitor of oocyte meiosis [20]. However, another research study demonstrated that oocyte meiosis is not affected by AMH [21]. It has been indicated that AMH did not have a significant effect on the IVM rate of cumulus-oocyte complexes (COCs), but could increase the blastocyst rate [22]. It can be seen that AMH plays an important role in the ovary; however, studies on its regulation of oocyte maturation rate are scarce, and the results are inconsistent, greatly limiting the application of AMH in female animals. Therefore, the present study was conducted to investigate the role and mechanism of AMH in regulating oocyte maturation and cumulus cell expansion in sheep in vitro, using the COCs as the target.

In this study, we analyzed the effects of AMH and FSH on cortical granule migration, mitochondrial distribution, the mitochondrial membrane potential (∆Ψm) and cytoskeletal structure in sheep oocytes. Through transcriptomic techniques, we sought to elucidate the mechanisms by which AMH impacts oocytes. The findings of this study will contribute to establishing an efficient method for enhancing the developmental potential of sheep oocytes.

## 2. Results

### 2.1. Effects of Different Concentrations of AMH Treatment on the Maturation Rate of Sheep Oocytes

As shown in Table 1, the maturation rate of the 100 ng/mL AMH group (81.52 ± 4.18%) was higher than that of the control group (71.36 ± 2.57%), 10 ng/mL AMH group (73.25 ± 3.18%), and 1000 ng/mL AMH group (58.46 ± 4.32%, *p* < 0.05).

### 2.2. Effect of AMH on the Maturation and Developmental Ability of Sheep Oocytes

**Figure 1:**
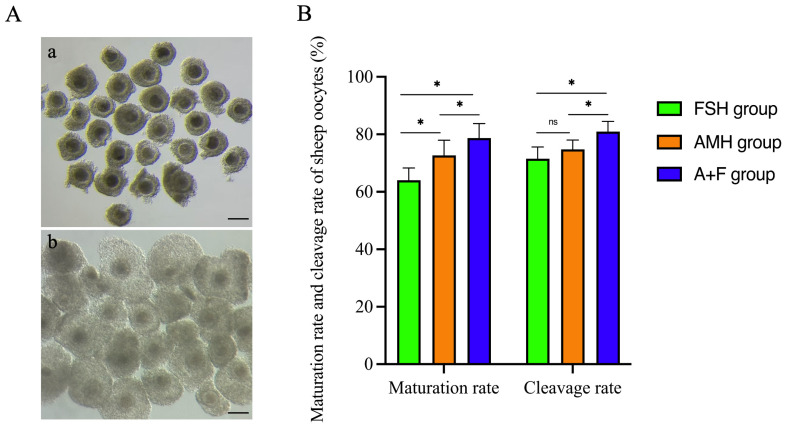
Effect of anti-Müllerian hormone (AMH) on the maturation rate of sheep oocytes. (**A**) Representative images. a: immature oocytes; b: mature oocytes. Scale bar = 100 μm. (**B**) The maturation rate and cleavage rate of sheep oocytes in different treatment groups. * *p* < 0.05; ns, no significant difference *p* ≥ 0.05.

Figure 1B shows that the maturation rate of the A+F group (86.49 ± 3.03%) was higher than that of the FSH group (67.19 ± 3.77%) and AMH group (79.13 ± 4.42%, *p* < 0.05). The cleavage rate of the A+F group was higher than that of the FSH group and AMH group, and there were no significant differences between the FSH group and AMH group.

### 2.3. Effect of AMH on the Viability of Sheep Oocytes

**Figure 2:**
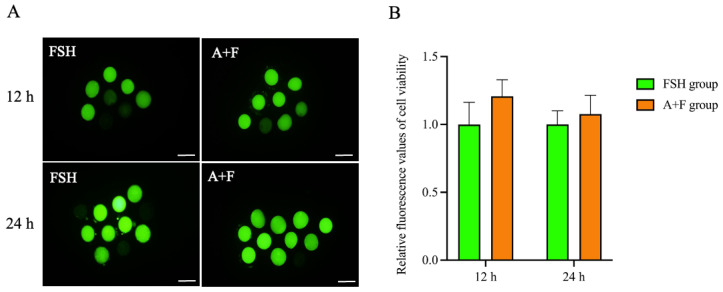
Effect of AMH on sheep oocytes viability. (**A**) Fluorescence images of oocyte cell viability in FSH and A+F groups at 12 h and 24 h. Scale bar = 100 μm. (**B**) The relative fluorescence values of oocyte viability in different treatment groups. Data were presented as the mean ± SD from at least three independent experiments.

Figure 2 shows the viability of sheep oocytes cultured for 12 h. Compared with the FSH group, the viability of oocytes in the A+F group was higher, but the difference was not significant (*p* > 0.05). There was no significant difference in the activity of sheep oocytes between the A+F group cultured for 24 h and the FSH group (*p* > 0.05).

### 2.4. Effect of AMH on the Cytoskeleton of Sheep Oocytes

**Figure 3:**
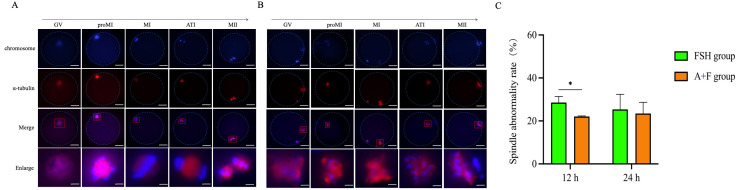
Effect of AMH supplementation during IVM on cytoskeleton in sheep oocytes. (**A**) Fluorescence images of a normal spindle in oocytes. (**B**) Fluorescence images of an abnormal spindle in oocytes. Scale bar = 20 μm. (**C**) The spindle abnormality rate of oocytes in different treatment groups. Data were presented as the mean ± SD from at least three independent experiments. * *p* < 0.05.

The spindle tissue and chromosome arrangement of oocytes at 12 h and 24 h were analyzed by α-tubulin fluorescence staining and Hoechst 33342 staining. The results are shown in Figure 3. The abnormal spindle rate of oocytes in the AMH group was substantially lower than that of the FSH group. The specific manifestation was that the spindle distortion rate of oocytes added with 12 h of AMH was significantly lower than that of the FSH group (*p* < 0.05). Compared with the FSH group, although the difference was not significant in the A+F group, the spindle distortion rate was still lower than that in the FSH group, as shown by the experimental results. It has been confirmed that the addition of AMH can maintain the normal morphology of the spindle in sheep oocytes.

### 2.5. Effect of AMH on the CGs Migration in Sheep Oocytes

**Figure 4:**
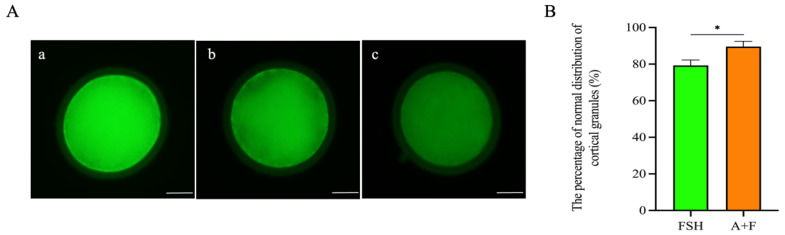
Effect of AMH on the CGs migration in sheep oocytes. (**A**) Representative images. a: The CGs are evenly distributed; b: The CGs are not evenly distributed; c: The CG signals are weak. Scale bar = 20 μm. (**B**) The percentage of normal distribution of CGs in oocytes of different treatment groups. Data were presented as the mean ± SD from at least three independent experiments. * *p* < 0.05.

To evaluate the effect of AMH treatment on the CGs of oocytes, FITC-PNA was used for immunofluorescence staining of the CGs of oocytes. As shown in Figure 4, the percentage of normal distribution of CGs in the FSH group was significantly lower than that of the A+F group (*p* < 0.05). It indicates that AMH can enhance the cytoplasmic maturation of oocytes by increasing the dynamic distribution of cortical granules.

### 2.6. Effect of AMH on the Distribution of Mitochondrial and ΔΨm in Sheep Oocytes

**Figure 5:**
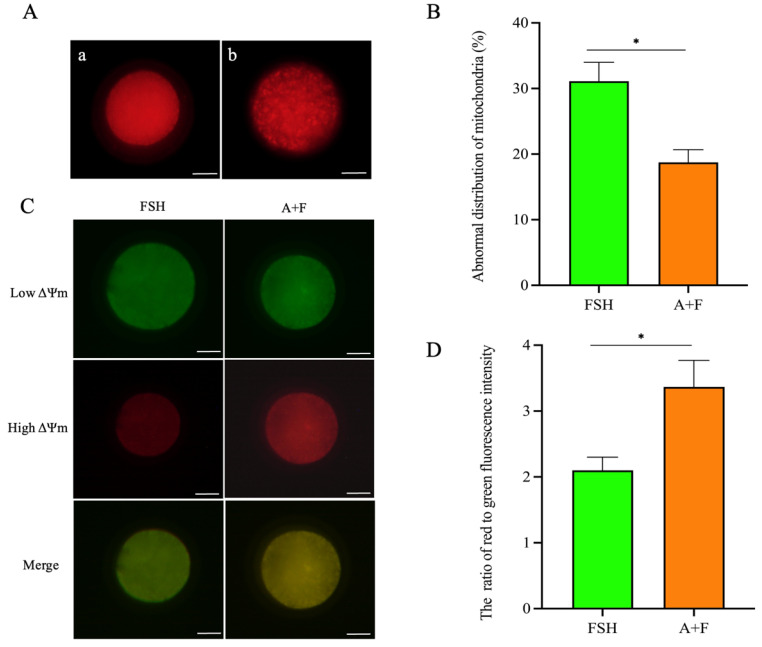
Effect of AMH supplementation during IVM on mitochondria distribution and mitochondrial membrane potential (ΔΨm) in sheep oocytes. (**A**) Representative images of sheep oocytes MitoTracker staining. a: The mitochondria are evenly distributed; b: The mitochondria are not evenly distributed. Scale bar = 20 μm. (**B**) The percentage of abnormal distribution of mitochondria in oocytes of different treatment groups. (**C**) Representative images of JC-1 staining in sheep oocytes. Green: JC-1 monomeric form (low ΔΨm); Red: JC-1-aggregated form (higher ΔΨm). Merge: merging of images with green and red fluorescence. Scale bar = 20 μm. (**D**) The ratio of red to green fluorescence intensity in oocytes of different treatment groups. Data were presented as the mean ± SD from at least three independent experiments. * *p* < 0.05.

As shown in Figure 5B, compared with the FSH group, the abnormal distribution rate of mitochondria in the A+F group was significantly decreased (*p* < 0.05), indicating that adding 100 ng/mL of AMH can promote cytoplasmic maturation of sheep oocytes by regulating mitochondrial biosynthesis. Then, we measured oocyte ΔΨm using JC-1 staining, with high-membrane-potential mitochondria exhibiting red fluorescence and low-membrane-potential mitochondria displaying green fluorescence. As shown in Figure 5D, the ΔΨm of the A+F group was significantly higher compared to the FSH group (*p* < 0.05).

### 2.7. Screening of Differentially Expressed Genes (DEGs) in AMH-Treated Sheep Oocytes

**Figure 6:**
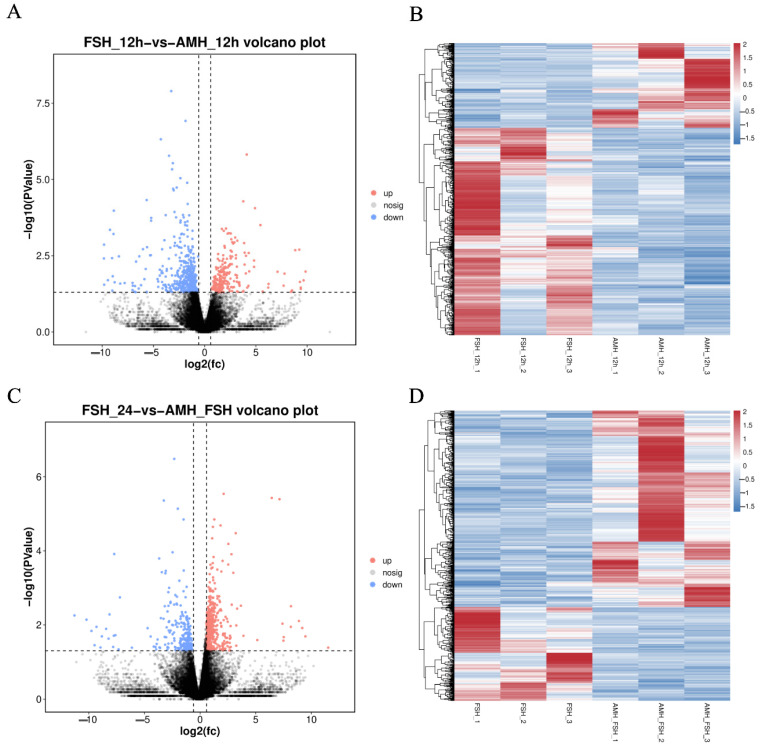
Differential gene expression analysis. (**A**) FSH_12 h-vs-AMH_12 h volcano plot of DEGs; (**B**) FSH_12 h-vs-AMH_12 h histogram of DEGs; (**C**) FSH_24 h-vs-AMH_FSH volcano plot of DEGs; (**D**) FSH_24 h-vs-AMH_FSH histogram of DEGs.

The IVM effect is better when the medium is changed 12 h after AMH addition. Therefore, we specifically focus on the gene expression regulation of oocytes after 12 h of both FSH and AMH addition. Figure 6A–D illustrates comparative analyses of DEGs in sheep oocytes. A total of 741 DEGs were between the FSH_12 h and AMH_12 h groups, including 215 up-regulated genes and 526 down-regulated genes, such as hormone regulation (*FST*, *CYP11A1*), oocyte maturation-related genes (*INHBE*, *IHH*), antioxidant gene (*GSTA1*), mitochondrial-related gene (*LOC114112897*), and cytoskeletal gene (*RAC2*).

A total of 746 DEGs were between the FSH_24 h and A+F groups, including 505 up-regulated genes and 241 down-regulated genes, such as the cytoskeleton-related genes (*TUBB6*, *EZR*), antioxidant gene (*GSTA1*), cumulus cells related gene (*FOS*), hormone related genes (*STAR*, *ESR2*), cell cycle and meiosis-related genes (*CUL1*, *BARD1*), and metabolism regulation related gene (*TDO2*).

### 2.8. GO Enrichment Analysis of the DEGs

**Figure 7:**
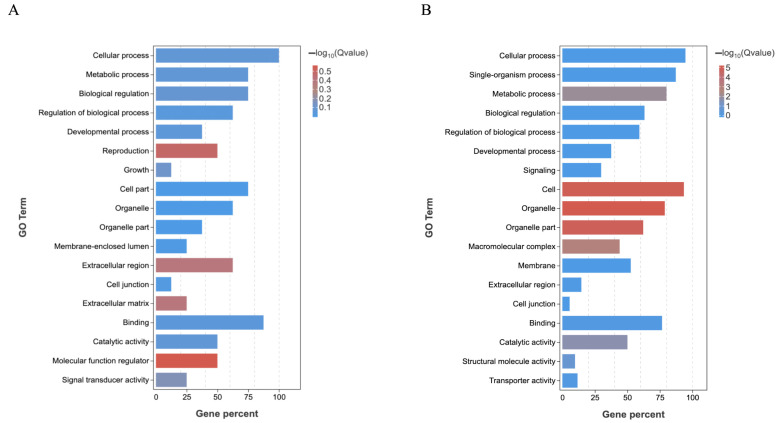
GO term enrichment for DEGs in the FSH_12 h-vs-AMH_12 h comparison. (**A**) FSH_12 h-vs-AMH_12 h GO term enrichment for DEGs; (**B**) FSH_24 h-vs-AMH_FSH GO term enrichment for DEGs.

Figure 7A shows the GO terms with a significant enrichment of DEGs between the FSH_12 h and AMH_12 h groups. The GO enrichment analysis revealed that biological processes (BPs) mainly consisted of biological regulation, development process and growth. Cell components (CCs) primarily involve the organelle, membrane and cell junction. Molecular function (MF) was primarily associated with binding, molecular function regulator, and signal transducer activity. Figure 7B shows the GO terms with a significant enrichment of DEGs between the FSH_24 h and AMH_24 h groups. The GO enrichment analysis revealed that BP mainly consisted of cellular processes, single-organism process and signaling. CCs primarily involve the organelle part, the macromolecular complex and the membrane. MF was primarily associated with binding, catalytic activity, and structural molecule activity.

### 2.9. Significantly Regulated Biological Signaling Pathways in Response to AMH Treatment in Sheep Oocytes

**Figure 8:**
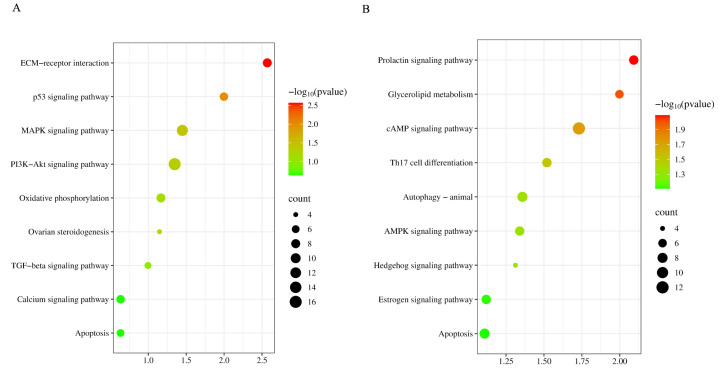
Bubble chart of the pathways in KEGG analysis of DEGs. (**A**) FSH_12 h-vs-AMH_12 h KEGG analysis of DEGs; (**B**) FSH_24 h-vs-AMH_FSH KEGG analysis of DEGs.

Figure 8A illustrates significantly enriched KEGG pathways between the FSH_12 h and AMH_12 h groups, such as p53, MAPK, PI3K-Akt and TGF-beta signaling pathways. Figure 8B illustrates significantly enriched KEGG pathways between the FSH_12 h and AMH_24 h groups, such as cAMP, AMPK, Hedgehog and estrogen signaling pathways.

### 2.10. Effect of AMH on Gene Expression in Sheep Oocytes

**Figure 9:**
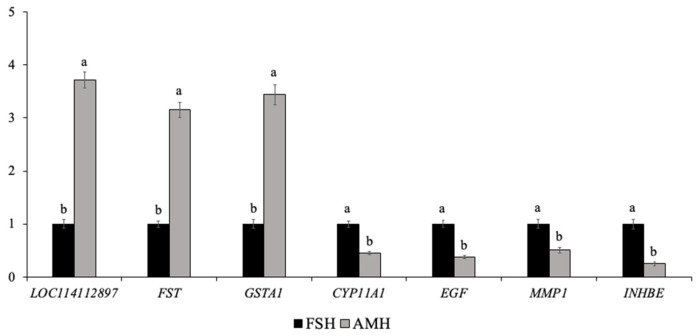
Effect of AMH on gene expression in sheep oocytes. ^a,b^ Values with different superscripts indicate significant difference between groups (*p* < 0.05).

As shown in Figure 9, the mRNA expression levels of gene (*LOC114112897*), gene (*FST*), and oxidative stress gene (*GSTA1*) in the oocytes of the AMH group were significantly higher than those of the control group. The mRNA expression levels of *CYP11A1*, *EGF*, *MMP1* and *INHBE* in the AMH group were lower than that of the control group (*p <* 0.05).

## 3. Discussion

Research data indicate that AMH may directly act on oocytes and influence their maturation process [23,24]. In this study, we explored the optimal concentration of AMH in sheep oocytes by adding AMH alone to the IVM medium, and found that the highest maturation rate of AMH was obtained with the addition of 100 ng/mL, which is the same as the results of the study by Zhang on mouse oocytes [22]. Studies have shown that AMH could inhibit the promoting effect of FSH on the in vitro maturation of COCs, indicating that FSH and AMH have antagonistic effects and should not be used together in the maturation medium [25,26]. In the present study, the maturation rate and cleavage rate of the A+F group were both higher than those of the FSH group and AMH group. It was found that the sequential addition of AMH and FSH during maturation induced the maturation and quality of sheep oocytes, and the developmental ability was higher than that of AMH and FSH added alone. This result is similar to the trends observed in mice, where AMH treatment enhanced the quality and development capacity of oocytes [27].

Activity staining of oocytes was performed to determine whether there was an inhibitory effect of AMH activity on oocytes. The results of the study showed that the cellular activity was higher in the AMH-treated group than in the FSH-alone-treated group for both 12 h and 24 h treatments, although the differences between the groups were not significant. It indicates that AMH does not affect the activity of oocytes. Data from some studies may indirectly support the results of the present study that AMH will target to attenuate the stimulatory effect of FSH in granulosa cells and will not affect the basal expression of genes [28].

Normal rearrangement of the spindle is one of the most important indicators of oocyte maturation [29]. In this experiment, the spindle organization and chromosome arrangement of sheep oocytes from different treatment groups at 12 h and 24 h were analyzed based on fluorescent staining results. The results revealed that the spindle aberration rates of oocytes with AMH added were lower than those of the FSH group, and the results at 12 h were significant, suggesting that AMH may improve the maturation rate of oocytes by preventing chromosome misalignment and spindle disorganization in oocytes.

CGs are organelles derived from the Golgi apparatus, which are distributed in the cortical layer of oocytes and are anchored to the cortical cytoskeleton composed of filamentous actin [30]. The CGs play a crucial role in the cytoplasmic maturation process of mammalian oocytes and in preventing multiple sperm entry into the oocyte after fertilization [31,32]. The results of this experiment indicate that the distribution of CGs in the sheep oocytes treated with AMH + FSH was significantly higher than that in the FSH group, suggesting that AMH can enhance the cytoplasmic maturation of oocytes by increasing the dynamics of CGs.

Mitochondrial function is one of the key factors affecting oocyte quality [33], whose dysfunction leads to abnormal oocyte meiosis and maturation arrest [34]. It has been shown that with significant structural changes in the distribution of mitochondria occurring as the oocyte nucleus matures [35], abnormal mitochondrial distribution disrupts oocyte cytoplasmic function and calcium homeostasis [36]. At the same time, the decrease in mitochondrial membrane potential will damage mitochondrial function and have an adverse effect on the maturation of oocytes [37]. In the present study, we found that the addition of AMH increased the uniform distribution of mitochondria and the level of ΔΨm in sheep oocytes, suggesting that AMH may promote cytoplasmic maturation of sheep oocytes by regulating mitochondrial biosynthesis. This is similar to previous findings, which indicated that mitochondrial function is the most influential factor in AMH initiation [38], and that active mitochondria, mitochondrial DNA content, and adenosine triphosphate levels were more abundant in oocytes and embryos isolated from AMH-initiated animals compared to control animals.

In this study, we compared transcriptomics of the AMH and control group oocytes using Smart-seq2 scRNA-seq technology, and verified the expression levels of the DEGs through RT-qPCR. *CYP11A1* is one of the key enzymes in steroid hormone synthesis, primarily responsible for converting cholesterol into pregnenolone, which is then converted into P4 under the catalysis of 3β-HSD [39]. From mammalian studies, AMH is believed to inhibit the steroid production induced by FSH [40]. Our study revealed that AMH significantly reduced the expression level of *CYP11A1*, which is consistent with previous findings that AMH reduces the expression of aromatase and *CYP11A1* [28,41]. The protein encoded by the *LOC114112897* (*NDUFC1*) gene is a subunit of mitochondrial respiratory chain complex I, which is involved in cellular respiration and is critical for maintaining mitochondrial function and energy metabolism [42]. Its upregulation may inhibit apoptosis by maintaining mitochondrial function and reducing the accumulation of reactive oxygen species (ROS) [43]. *GSTA1* protects oocytes from oxidative damage, maintains mitochondrial membrane potential, and supports steroid hormone synthesis to promote oocyte maturation [44]. Our results revealed that AMH treatment increased the *LOC114112897* and *GSTA1* expression, suggesting that AMH can improve mitochondrial function and decrease the level of ROS in oocytes, thereby promoting the maturation of oocytes.

The enrichment analysis of DEGs revealed distinct significant pathways at different culture stages. At 12 h, pathways, including the p53, MAPK, PI3k-Akt, and TGF-beta signaling pathways, are overrepresented, whereas at 24 h, pathways such as cAMP, autophagy, AMPK, Hedgehog, estrogen signaling pathways are underrepresented. AMH modulates oocyte maturation mainly by regulating the activity of these cascades in a time-dependent manner. The p53 pathway is a master regulator of various cellular processes, including apoptosis, cell cycle arrest, senescence, DNA repair [45], and steroid hormone regulation [46]. The altered p53 signaling induced by AMH may further affect the physiological state of maturing oocytes. Activation of MAPK promotes the transition of oocytes from metaphase to anaphase of meiosis and participates in the regulation of microtubule organization and meiotic spindle assembly [47]. Activation of the PI3K-Akt signaling pathway promotes the resumption and maturation of oocyte meiosis [48].

The cAMP signaling pathway maintains oocyte arrest at metaphase of meiosis by activating PKA [49]. The AMPK signaling pathway promotes polar body formation and inhibits premature activation during oocyte meiosis maturation [50]. The loss of its activity induces organelle dysfunction and oxidative stress during oocyte senescence [51]. This indicates that oocyte development and maturation are complex and delicate processes involving the collaborative action of multiple signaling pathways. Nevertheless, further functional experiments are still required to clarify the detailed upstream and downstream molecular interactions among these signaling cascades.

## 4. Materials and Methods

All chemicals and reagents used were purchased from Sigma-Aldrich Chemical Company (St. Louis, MO, USA) unless otherwise stated.

### 4.1. In Vitro Maturation (IVM) of Oocytes

Sheep ovaries were collected from the slaughterhouse and transported to the laboratory within 2 h at 30–35 °C. The ovaries were placed in a 10 mm dish with washing solution, the ovaries were clamped with tweezers, and the follicles were cut with a scalpel to obtain COCs. COCs were washed, and those with at least three layers of cumulus cells were chosen for the experiment. The basic IVM medium contained TCM199 (Gibco BRL, Carlsbad, CA, USA) supplemented with 10% estrus sheep serum (ESS, Hyclone; Gibco BRL), 1 µg/mL estradiol, and 10 µg/mL luteinizing hormone (LH). The experiment groups are as follows: (1) control group: the basic IVM medium for 24 h; (2) FSH group: 10 µg/mL FSH supplementation in the basic IVM medium for 24 h; (3) AMH group: AMH supplementation in the basic IVM medium for 24 h; and (4) A+F group: AMH supplementation in the basic IVM medium for 12 h and then transferred to the FSH supplementation in the basic IVM medium for 12 h.

### 4.2. In Vitro Fertilization (IVF) and In Vitro Vulture (IVC)

After IVM, oocytes were washed three times with BO-IVF (IVF Bioscience, Falmouth, UK). The frozen sheep sperm was thawed at 37 °C for 15 s and then rinsed twice at 600× *g* for 5 min in the BO-SemenPrep (IVF Bioscience, Falmouth, UK) medium. The sperm density was adjusted to 1 × 10^6^ sperm/mL and then incubated with oocytes at 38.5 °C in humidified air containing 5% CO_2_ for 8–18 h. After IVF, the zygotes were cultured in BO-IVC (IVF Bioscience, Falmouth, UK) at 38.5 °C in humidified air containing 5% CO_2_. On day 3 and day 7, the cleavage rate and blastocyst rate were respectively counted.

### 4.3. Oocyte Viability

The oocyte viability was assessed with fluorescein diacetate (FDA) (IF0160, Solarbio, Beijing, China) staining according to the method previously described by Mohr et al., 1980 [52]. Each group has at least three replicates and 35 oocytes each time. Briefly, oocytes were washed twice with PBS containing 0.1% polyvinyl alcohol (PVA) and then incubated in 5 μg/mL FDA for 30 min at 37 °C in a humidified 5% CO_2_. Finally, oocytes were observed on the epifluorescence inverted microscope (Nikon, Tokyo, Japan), and the fluorescence intensity was determined using ImageJ software version 1.8.0 (NIH, Bethesda, MD, USA).

### 4.4. Mitochondrial Distribution

The mitochondrial distribution was detected according to the method described by Zhao et al., 2018 [53]. The oocytes were fixed and washed three times with PBS. Subsequently, the oocytes were incubated in 200 nM Mitotracker Green (M7514; Thermo Fisher Scientific, Waltham, MA, USA) for 30 min at 37 °C in 5% CO_2_, washed in DPBS with 0.1% PVA, and examined by the epifluorescence inverted microscope (Nikon, Tokyo, Japan). At least 35 oocytes were checked for each group, and at least three replicates were conducted for each group.

### 4.5. Mitochondrial Membrane Potential (ΔΨm) Examination of Oocytes

The ΔΨm level in oocytes of each group was detected using the Mitochondrial Membrane Potential Assay Kit (Beyotime, Shanghai, China) according to the instructions. Each group was built up with at least 35 oocytes and at least three replicates. First, the oocytes were washed three times in 0.1% PVA/PBS solution, and then put into 10 μg/mL JC-1 staining solution, and stained for 30 min at 38.5 °C in 5% CO_2_. Subsequently, the oocytes were washed with 0.1% PVA/PBS, and then an epifluorescence inverted microscope (Nikon, Tokyo, Japan) was used for scanning [54]. The fluorescence intensity of each oocyte was analyzed using ImageJ software version 1.8.0 (NIH, Bethesda, MD, USA), and the fluorescence value of the acquired fluorescence images was counted, and the ratio of the intensity of red fluorescence to green fluorescence was ΔΨm.

### 4.6. Oocyte Cytoskeleton Staining

Staining was performed with at least 35 oocytes for each group and at least three replicates. According to a previous method described by Cai et al., 2021 [55], the oocytes were fixed with 4% paraformaldehyde for 30 min and then washed with 0.1% TritonX-100 for 10 min. The oocytes were treated with Tubulin-Tracker Red solution (C1050, Beyotime, Shanghai, China) for 30 min in the dark at 38.5 °C in 5% CO_2_. Then, they were washed three times with 0.1% PVA/PBS and incubated in 1 mg/mL DAPI at 37 °C for 5 min. Finally, they were examined with an epifluorescence inverted microscope (Nikon, Tokyo, Japan). ImageJ software version 1.8.0 (NIH, Bethesda, MD, USA) was utilized for the fluorescence intensity of oocytes.

### 4.7. Distribution of Cortical Granules (CGs) in Oocytes

Minor modifications were made based on the method described by Hao et al., 2022 [56]. Each group (35 oocytes) had at least three replicates. Oocytes were fixed with 4% paraformaldehyde for 30 min and then treated with 0.1% TritonX-100 in PBS for 10 min. The oocytes were washed three times with 0.3% BSA in PBS for 5 min each time and then treated with 10 μg/mL fluorescein isothiocyanate peptide nucleic acid (FITC-PNA) (L-7381; Sigma-Aldrich) for 30 min in the dark at 38.5 °C in 5% CO_2_. After that, they were washed three times with 0.1% PVA/PBS and examined with an epifluorescence inverted microscope (Nikon, Tokyo, Japan). The fluorescence intensity of oocytes was measured using the ImageJ software version 1.8.0 (NIH, Bethesda, MD, USA).

### 4.8. Sample Preparation, Library Construction and RNA Sequencing

Oocytes were washed and pooled in lysis buffer, and the Smart-seq2 scRNA-seq library was prepared following the protocol described by Treger et al., 2019 [57] with minor adjustments. Total RNA isolation from experimental samples was accomplished utilizing the Universal RNA Extraction CZ Kit (RNC643, ONREW, Guangzhou, Guangdong, China) in compliance with the manufacturer’s protocols. The synthesis and amplification of cDNA were implemented with the Single Cell Full Length mRNA-Amplification Kit (N712-30, Vazyme, Jiangsu, China), followed by purification utilizing VAHTS DNA Clean Beads (N411-02, Vazyme, Jiangsu, China). Library construction for DNA sequencing was performed referring to the official operation guide of Vazyme TruePrep DNA Library Prep Kit V2 (TD503-02, Vazyme, Jiangsu, China). Qualified libraries were sequenced on an Illumina Novaseq 6000 with a PE150 strategy, generating 150-bp reads from both ends.

### 4.9. Bioinformatics Analysis

In this research, Gene Ontology (GO) enrichment analysis was conducted with a focus on three core categories: biological processes, cellular components and molecular functions, drawing on data from the official GO database (http://www.geneontology.org/). Meanwhile, the Kyoto Encyclopedia of Genes and Genomes (KEGGs) enrichment analysis was employed to identify biological pathways with significant enrichment. Significance was set at a *p*-value of <0.05.

### 4.10. Quantitative Real-Time Polymerase Chain Reaction (qRT-PCR) of Genes

The relative mRNA expression levels in oocytes were quantified using the Single Cell-to-CT qRT-PCR Kit (Life Technologies, Carlsbad, CA, USA). Amplification was carried out on an ABI 7500 SDS system (Applied Biosystems, Foster City, CA, USA) under the following cycling conditions: initial denaturation at 95 °C for 2 min, followed by 40 cycles of 95 °C for 10 s and 60 °C for 30 s. Gene expression fold changes were calculated via the comparative Ct (2^−ΔΔCt^) method, with *β-actin* serving as the internal reference control. The primer sequences for all candidate genes are provided in Table 2.

### 4.11. Statistical Analysis

All experiments were repeated at least three times, and all data were presented as mean ± standard deviation (SD). All analyses were performed using SAS software version 9.2.0 (SAS Institute, Cary, NC, USA) to compare the groups. Differences were regarded to be significant when *p* < 0.05.

## 5. Conclusions

In the context of our investigation, the sequential use of AMH and FSH produces a sequential effect, significantly improving the in vitro maturation rate and quality of oocytes. Likewise, the A+F group significantly increased the normal distribution rate of cortical granules and mitochondrial function, while reducing the rate of spindle abnormalities in sheep oocytes. These findings indicate that the sequential use of AMH and FSH optimizes oocyte maturation, providing crucial evidence for developing more efficient IVM culture systems.

## Figures and Tables

**Table 1:** Effect of different concentrations of anti-Müllerian hormone (AMH) on the maturation ability of sheep oocytes.

Groups	No. COCs	Maturation Rate
Control (0 ng/mL AMH)	112	71.36 ± 2.57% ^b^
10 ng/mL AMH	110	73.25 ± 3.18% ^b^
100 ng/mL AMH	111	81.52 ± 4.18% ^a^
1000 ng/mL AMH	110	58.46 ± 4.32% ^c^

^a,b,c^ Values with different superscripts represent significant differences between groups (*p* < 0.05).

**Table 2:** Primers used for candidate genes in sheep oocytes.

Gene	Primers (5′-3′)	Size (bp)	GenBank Accession No.
*LOC114112897*	F: CCTTTTTGCGCCCATTCTGG	111	XM_027963981.2
	R: GCCAGTTAGGACTGCCATGT		
*FST*	F: GGATCTTGCAACTCCATTTCG	117	XM_012096672.4
	R: CACTGAACATTGGTGGAGGGT		
*GSTA1*	F: TATTCAGAGGGGGTGGCAGA	148	XM_027958329.3
	R: CGTGGCTCTTCAGCACATTT		
*CYP11A1*	F: GGTCCCATTTACAGGGAGAAGC	169	XM_060401375.1
	R: TAAACAGGACTCCGATGGGT		
*EGF*	F: GTGAGATGGGTGTCCCAGTG	128	NM_001178130.3
	R: GGGTGGAGTAGAGTCAAGACAG		
*MMP1*	F: CAGGCCATCTATGGACCTTCC	169	XM_012095308.4
	R: CAACGTCTGCGTAGAAGGGA		
*INHBE*	F: TGCAGTCCTCACAGACTCCTC	87	XM_004006544.5
	R: TGATACAGGTGATGGGACCG		
*β-actin*	F: ATGAGTCTGGCCCCTCCATT	137	XM_004013078.5
	R: CATGAGGCTAGCATGAGGTG		

## Data Availability

The original contributions presented in this study are included in the article. Further inquiries can be directed to the corresponding authors.
